# Effect of carbohydrate feeding on the bone metabolic response to running

**DOI:** 10.1152/japplphysiol.00241.2015

**Published:** 2015-08-06

**Authors:** Craig Sale, Ian Varley, Thomas W. Jones, Ruth M. James, Jonathan C. Y. Tang, William D. Fraser, Julie P. Greeves

**Affiliations:** ^1^Musculoskeletal Physiology Research Group, Sport, Health and Performance Enhancement Research Centre, School of Science and Technology, Nottingham Trent University, Nottingham, United Kingdom;; ^2^Institute of Neuroscience, Newcastle University, Newcastle upon Tyne, United Kingdom;; ^3^Norwich Medical School, University of East Anglia, Norwich, United Kingdom;; ^4^Norfolk and Norwich University Hospital, Norwich, United Kingdom; and; ^5^Department of Occupational Medicine, HQ Army Recruiting and Training Division, Upavon, United Kingdom

**Keywords:** carbohydrate, feeding, bone metabolism, running, exercise

## Abstract

Bone resorption is increased after running, with no change in bone formation. Feeding during exercise might attenuate this increase, preventing associated problems for bone. This study investigated the immediate and short-term bone metabolic responses to carbohydrate (CHO) feeding during treadmill running. Ten men completed two 7-day trials, once being fed CHO (8% glucose immediately before, every 20 min during, and immediately after exercise at a rate of 0.7 g CHO·kg body mass^−1^·h^−1^) and once being fed placebo (PBO). On *day 4* of each trial, participants completed a 120-min treadmill run at 70% of maximal oxygen consumption (V̇o_2 max_). Blood was taken at baseline (BASE), immediately after exercise (EE), after 60 (R1) and 120 (R2) min of recovery, and on three follow-up days (FU1-FU3). Markers of bone resorption [COOH-terminal telopeptide region of collagen type 1 (β-CTX)] and formation [NH_2_-terminal propeptides of procollagen type 1 (P1NP)] were measured, along with osteocalcin (OC), parathyroid hormone (PTH), albumin-adjusted calcium (ACa), phosphate, glucagon-like peptide-2 (GLP-2), interleukin-6 (IL-6), insulin, cortisol, leptin, and osteoprotogerin (OPG). Area under the curve was calculated in terms of the immediate (BASE, EE, R1, and R2) and short-term (BASE, FU1, FU2, and FU3) responses to exercise. β-CTX, P1NP, and IL-6 responses to exercise were significantly lower in the immediate postexercise period with CHO feeding compared with PBO (β-CTX: *P* = 0.028; P1NP: *P* = 0.021; IL-6: *P* = 0.036), although there was no difference in the short-term response (β-CTX: *P* = 0.856; P1NP: *P* = 0.721; IL-6: *P* = 0.327). No other variable was significantly affected by CHO feeding during exercise. We conclude that CHO feeding during exercise attenuated the β-CTX and P1NP responses in the hours but not days following exercise, indicating an acute effect of CHO feeding on bone turnover.

feeding influences the circadian rhythm of bone metabolism at rest (21). Markers of bone resorption decrease after mixed-meal feeding (3) and ingestion of individual macronutrients at rest (1, 7). Reductions in bone resorption occur after oral but not parenteral administration of glucose, suggesting that enteric hormones might play a part in mediating the effects of glucose on bone metabolism (1). This is supported by studies showing that administration of octreotide, an inhibitor of enteric hormone secretion, abolishes the reduction in bone resorption following glucose ingestion (4). These findings suggest a potential means to modulate bone turnover by nutritional strategies or through manipulation of dietary composition (25).

Exhaustive weight-bearing exercise increases bone resorption by 40–45% for up to four consecutive days without a concomitant increase in bone formation, potentially resulting in a short-term net loss of bone (19). Attenuating bone resorption is a potential countermeasure to the bone uncoupling shown to occur with exercise that might predispose athletes and military recruits to stress fractures and other associated injuries (26). Feeding practices, before, during, and after exercise, influence the interaction between exercise and bone turnover, making them potentially important for offsetting bone loss. Scott et al. (22) showed that feeding a mixed meal prior to exercise reduced COOH-terminal telopeptide region of collagen type 1 (β-CTX) concentrations before a 60-min treadmill run, although the subsequent increase in β-CTX during exercise was similar in fed and fasted groups. This indicated a novel interaction between feeding, exercise, and bone metabolism that requires further study, particularly in relation to feeding practices during and after exercise.

Carbohydrate (CHO), fat, and protein all decrease β-CTX (3, 4); CHO ingestion during exercise is an established nutritional practice for athletes, proven to enhance physical performance and exercise capacity by providing additional fuel to the muscle (11). A recent study (5) suggests that CHO is a candidate nutrient for modulating bone resorption during an exercise program (8-day intensive training), although factors that may have mediated these responses were not examined.

The aim of this study was to investigate the responses of bone metabolism to CHO feeding during exercise in the hours (immediate) and days (short term) following a single bout of strenuous treadmill running. We also measured markers associated with feeding, exercise, and bone to explore possible mediating and mechanistic factors.

## METHODS

### Participants

Ten healthy, physically active men [means ± SD: age 24 ± 3 yr, height 1.75 ± 0.08 m, body mass 72.9 ± 7.5 kg, body fat percentage 14.3 ± 2.4%, maximal oxygen consumption (V̇o_2 max_) 53.0 ± 6.4 ml·kg^−1^·min^−1^] provided informed consent and completed medical history questionnaires. The institutional research ethics committee approved the study in accordance with the Declaration of Helsinki.

Participants were included if they were nonsmokers, had not suffered a bone fracture or injury of any type in the previous 12 mo, were free from musculoskeletal injury, were not taking any medication, and were not suffering from any condition known to affect bone metabolism. Eligibility of each participant was confirmed verbally and with a medical screening questionnaire.

### Experimental Design

All participants completed two randomized, repeated-measures, counterbalanced 7-day experimental trials, involving either placebo (PBO) or CHO ingestion during 120 min of treadmill running at 70% of V̇o_2 max_. Trials were separated by 14 days to allow participants to recover from blood sampling and dietary control and to allow bone marker concentrations to return to baseline values. Participants were required to refrain from exercise and caffeine and alcohol consumption for 48 h prior to trials. Participants recorded dietary intake during the first trial and repeated the same dietary pattern during the second trial to control for the influence of feeding on bone metabolism (25).

### Experimental Procedures

#### Preliminary measurements.

Height and body mass were recorded before body fat analysis was conducted by bioelectrical impedance (Bodystat 1500, Bodystat, Douglas, Isle of Man, UK). Preliminary testing also involved the assessment of the cardiorespiratory responses to running. Participants first performed a submaximal test to establish the relationship between running speed and oxygen consumption during level running, which was completed at a 0% gradient from a gentle starting speed. The speed of the treadmill was increased by 1 km/h at 3-min intervals for at least 15 min (5 stages). Expired air (∼1 min, inspiration to inspiration) was collected into Douglas bags during the last minute of each stage to determine oxygen consumption. Oxygen consumption at each stage of the submaximal test was plotted against the running speed at that stage so that the relationship between running speed and oxygen consumption could be determined.

The maximum oxygen uptake test was then performed after a 15-min recovery period, consisting of a continuous incremental uphill running test at constant speed until volitional exhaustion. Running speed for this test was determined from the results of the submaximal test. The gradient of the treadmill was increased by 1% at the end of each minute from a starting gradient of 0%. Once maximum oxygen uptake was determined, the oxygen consumption representing 70% V̇o_2 max_ was calculated. With the data from the submaximal running test, the running speed that elicited 70% V̇o_2 max_ at a 0% gradient was determined.

#### Trial days 1–3.

Participants adhered to their normal diet and refrained from exercise or strenuous physical exertion. Participants recorded their dietary intake and were asked about lifestyle activity (e.g., feelings of fatigue, sleep patterns), with diet and sleep patterns being replicated between trials.

#### Trial day 4.

Participants attended the laboratory (0800) after an overnight fast (since 2000 the previous evening) and remained fasted until the final blood sample was drawn. On arrival, nude body mass was determined and participants then rested in a semirecumbent position and were fitted with a heart rate monitor (Polar FS1, Polar Electro). At 0830, a resting blood sample (BASE) was withdrawn from a prominent forearm vein. Participants then completed 120 min of running on a treadmill (h/p/cosmos, Pulsar 4.0) at 70% V̇o_2 max_. Further blood samples were withdrawn immediately after exercise (EE) (1100) and after 60 (R1) (1200) and 120 (R2) (1300) min of recovery. Heart rate (HR) and rating of perceived exertion (RPE; 6–20 scale) were recorded before exercise and at 10-min intervals throughout exercise for participant monitoring purposes.

During the CHO trial, participants ingested an 8% glucose solution immediately before, every 20 min during, and immediately after exercise at a rate of 0.7 g CHO·kg body mass^−1^·h^−1^. The total amount of glucose ingested was 102.1 ± 10.6 g in a total solution volume of 1,276 ± 132 ml. These totals were divided equally over seven mean ingestions of 14.6 ± 1.5 g of glucose in 182 ± 19 ml of solution. During the PBO trial, participants ingested equal volumes of a taste-matched flavored water drink containing no CHO.

Upon completion of exercise, nude body mass was determined and participants consumed water equal to 150% of the body mass lost over the subsequent 120 min. Participants were instructed not to perform any further exercise.

#### Trial days 5–7.

Participants attended the laboratory (0800) after an overnight fast (from 2000 the previous evening) and rested for 30 min, after which a blood sample was withdrawn (0830) from a prominent forearm vein. During this time, participants continued to record their diet and maintained dietary control and refrained from all exercise.

### Storage and Analyses of Venous Blood

Fifteen milliliters of venous blood was dispensed into three 5-ml tubes lined with ethylenediaminetetraacetic acid (EDTA) and centrifuged immediately for 10 min at 2,000 *g* and 4°C. After centrifugation, plasma was dispensed into Eppendorf tubes and stored at −80°C for subsequent analyses of COOH-terminal telopeptide region of collagen type 1 (β-CTX), NH_2_-terminal propeptides of procollagen type 1 (P1NP), osteoprotogerin (OPG), osteocalcin (OC), parathyroid hormone (PTH), leptin, glucagon-like peptide-2 (GLP-2), and interleukin-6 (IL-6). Prior to storage, measurements of plasma glucose and lactate were performed (Yellow Springs Instruments 2300 STAT Plus, YSI).

β-CTX, P1NP, OC, and PTH were measured by electrochemiluminescent immunoassay (ECLIA) on a Modular Analytics E170 analyzer (Roche Diagnostics, Burgess Hill, UK). Interassay coefficient of variation (CV) for β-CTX was <3% between 0.2 and 1.5 μg/l, with sensitivity of 0.01 μg/l. P1NP interassay CV was <3% between 20 and 600 μg/l, with sensitivity of 8 μg/l. OC interassay CV was <5% between 2 and 200 μg/l, with sensitivity of 0.6 μg/l. PTH interassay CV was <4% between 1 and 30 pmol/l, with sensitivity of 0.8 pmol/l. OPG was measured with an enzyme-linked immunosorbent assay (ELISA) supplied by Immuno Diagnostic Systems (IDS, Boldon, UK), with an interassay CV of <8% across the range 1–30 pmol/l and sensitivity of 0.14 pmol/l. Leptin was measured by ELISA supplied by IDS, having an interassay CV of <8% across the range 3–50 μg/l and sensitivity of 1 μg/l. GLP-2 was measured by ELISA (Yanaihara Institute), with an interassay CV of 1.1–11.1% across the range 3.1–33.4 ng/ml and detection limit of 0.5 ng/ml. IL-6 was measured by ELISA (Quantikine HS, R&D Systems), with an interassay CV of <10% across the range 0.15–10 pg/ml and detection limit of 0.039 pg/ml.

The remaining 5 ml of venous blood was dispensed into a serum tube and allowed to clot at room temperature for 60 min before being centrifuged for 10 min at 2,000 *g* and 4°C. Resultant serum was dispensed into Eppendorf tubes and stored at −80°C for the subsequent analysis of cortisol, insulin, calcium, albumin, and phosphate (PO_4_).

Cortisol was measured with an ECLIA on the Roche Modular E170, with an interassay CV of <6% between 16 and 1,750 nmol/l and sensitivity of 8 nmol/l. Insulin was measured by ECLIA on a Cobas e601 (Roche Diagnostics, Burgess Hill, UK), having an interassay CV of <6.1% across the range 44–505 pmol/l and sensitivity of 1.8 pmol/l. Calcium, albumin, and PO_4_ were measured with standard commercial assays supplied by Roche Diagnostics performed on the Roche Modular E170. The range of measurement in serum is 0.05–5.00 mmol/l for calcium, 10–70 g/l for albumin, and 0.10–6.46 mmol/l for PO_4_.

### Statistical Analysis

Data are presented as means ± SD, and statistical significance was accepted at *P* ≤ 0.05. Data were analyzed with SPSS V20. Effects of exercise were assessed on the PBO trial data with a one-way ANOVA for normally distributed data and a Friedman's ANOVA for nonnormally distributed data. Within-exercise variables were analyzed with a repeated-measures ANOVA. The area under the curve (AUC) with respect to BASE was calculated for all biochemistry markers from the percent change data (27) for the immediate (BASE, EE, R1, and R2) and short-term (BASE, FU1, FU2, and FU3) responses to exercise. The two conditions were then compared with a paired-samples *t*-test for normally distributed data or a Wilcoxon's test for nonnormally distributed data.

## RESULTS

### BASE Biochemistry

Table 1 shows the mean ± SD concentrations for all variables. There were no differences between trials for any of the measures taken at BASE (*P* values from 0.143 to 0.990, data not shown).

**Table 1.** Concentrations of bone turnover markers, modulators of bone metabolism, and markers of calcium metabolism

	BASE	EE	R1	R2	FU1	FU2	FU3
*Markers of bone formation and resorption*							
β-CTX, ng/ml							
CHO	0.60 ± 0.23	0.43 ± 0.18	0.41 ± 0.20	0.39 ± 0.19	0.58 ± 0.20	0.60 ± 0.17	0.62 ± 0.21
PBO	0.54 ± 0.14	0.45 ± 0.17	0.45 ± 0.19	0.42 ± 0.16	0.56 ± 0.14	0.58 ± 0.19	0.61 ± 0.16
P1NP, ng/ml							
CHO	65.1 ± 28.7	73.8 ± 28.3	63.0 ± 24.2	65.7 ± 27.3	65.1 ± 26.0	68.4 ± 29.3	69.7 ± 29.2
PBO	63.1 ± 30.5	77.8 ± 34.9	68.3 ± 29.2	67.7 ± 25.8	67.9 ± 33.2	66.2 ± 31.7	68.4 ± 28.0
OC, ng/ml							
CHO	26.6 ± 10.8	29.7 ± 11.5	26.0 ± 9.7	24.0 ± 9.7	25.1 ± 9.1	26.0 ± 8.7	25.5 ± 9.3
PBO	25.1 ± 7.3	29.4 ± 6.6	25.9 ± 8.4	24.3 ± 7.2	22.5 ± 6.9	25.3 ± 9.2	25.6 ± 8.6
*Markers of calcium metabolism*							
PTH, pmol/l							
CHO	3.66 ± 0.68	7.13 ± 1.99	3.25 ± 1.41	3.25 ± 0.80	3.84 ± 0.89	3.78 ± 1.21	3.65 ± 0.55
PBO	3.66 ± 1.18	6.55 ± 1.97	3.55 ± 1.23	3.25 ± 1.15	3.85 ± 1.31	3.68 ± 1.37	4.26 ± 2.07
PO_4_, mmol/l							
CHO	1.17 ± 0.22	1.35 ± 0.19	1.01 ± 0.20	1.04 ± 0.11	1.24 ± 0.21	1.13 ± 0.15	1.14 ± 0.13
PBO	1.14 ± 0.11	1.36 ± 0.08	1.13 ± 0.20	1.04 ± 0.14	1.13 ± 0.15	1.02 ± 0.13	1.15 ± 0.15
ACa, mmol/l							
CHO	2.20 ± 0.11	2.24 ± 0.06	2.26 ± 0.21	2.18 ± 0.15	2.21 ± 0.09	2.12 ± 0.12	2.15 ± 0.06
PBO	2.15 ± 0.10	2.20 ± 0.11	2.12 ± 0.14	2.15 ± 0.14	2.13 ± 0.15	2.13 ± 0.14	2.14 ± 0.10
*Modulators of bone metabolism*							
Insulin, pmol/l							
CHO	68.2 ± 70.7	126.2 ± 99.0	46.0 ± 61.1	23.9 ± 18.5	66.0 ± 55.5	96.6 ± 163.3	81.4 ± 144.2
PBO	54.6 ± 33.1	63.5 ± 23.5	20.0 ± 15.4	22.0 ± 13.5	56.2 ± 66.0	111.7 ± 217.0	64.9 ± 68.0
Cortisol, nmol/l							
CHO	449.1 ± 113.5	478.9 ± 196.6	423.3 ± 188.9	354.2 ± 132.9	494.4 ± 119.1	454.8 ± 121.9	438.3 ± 133.0
PBO	485.7 ± 116.5	503.2 ± 80.5	500.1 ± 167.2	470.4 ± 139.6	469.6 ± 155.8	484.3 ± 136.8	468.8 ± 144.1
IL-6, pg/ml							
CHO	1.30 ± 0.61	12.17 ± 11.42	10.51 ± 7.96	7.54 ± 4.04	2.2 ± 1.74	1.58 ± 1.12	1.76 ± 1.33
PBO	1.09 ± 0.57	15.36 ± 9.36	11.14 ± 6.16	9.40 ± 3.04	1.56 ± 0.70	1.27 ± 0.47	1.10 ± 0.57
Leptin, ng/l							
CHO	3.91 ± 1.40	3.43 ± 1.41	2.95 ± 1.22	2.90 ± 1.24	3.90 ± 1.46	3.87 ± 1.20	4.09 ± 1.15
PBO	4.86 ± 2.74	4.08 ± 2.59	3.63 ± 2.77	3.42 ± 2.70	4.12 ± 2.82	4.30 ± 2.57	3.79 ± 0.61
OPG, pmol/l							
CHO	2.46 ± 1.28	3.16 ± 1.55	2.99 ± 0.85	3.30 ± 1.30	2.22 ± 0.87	2.22 ± 0.92	3.29 ± 1.62
PBO	2.55 ± 1.20	2.63 ± 1.05	2.48 ± 1.38	2.84 ± 1.37	2.17 ± 1.06	2.95 ± 1.02	2.73 ± 1.02
GLP-2, ng/ml							
CHO	8.28 ± 5.28	10.18 ± 6.52	8.47 ± 4.91	7.69 ± 4.61	9.22 ± 6.51	9.19 ± 5.30	9.08 ± 5.35
PBO	9.13 ± 6.44	9.29 ± 7.08	10.06 ± 7.52	9.09 ± 6.51	9.12 ± 6.05	8.29 ± 4.59	9.44 ± 5.83

Data are mean ± SD concentrations of bone turnover markers, modulators of bone metabolism, and markers of calcium metabolism at baseline (BASE), immediately after exercise (EE), during 2 h of recovery after exercise (R1, R2), and during 3 follow-up days (FU1–FU3) in the carbohydrate (CHO) and placebo (PBO) trials.

β-CTX, COOH-terminal telopeptide region of collagen type 1; P1NP, NH_2_-terminal propeptides of procollagen type 1; OC, osteocalcin; PTH, parathyroid hormone; ACa, albumin-adjusted calcium; IL-6, interleukin-6; OPG, osteoprotogerin; GLP-2, glucagon-like peptide-2.

### Exercise Variables

**Fig. 1.**
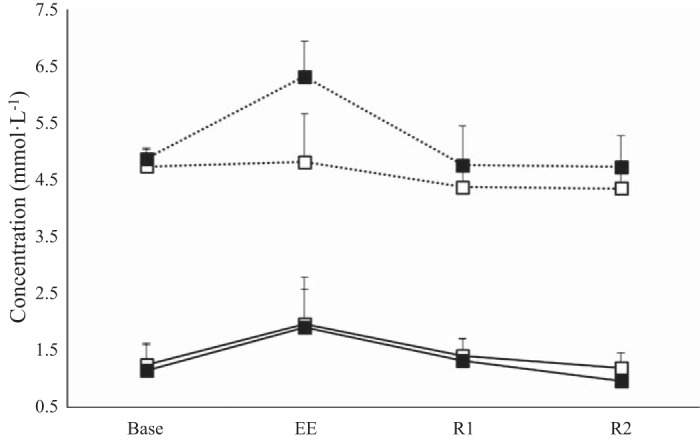
Blood glucose (dotted line) and blood lactate (solid line) concentrations in the carbohydrate (CHO; ■) and placebo (PBO; □) trials. Blood glucose concentration was significantly different between EE and all other time points and between CHO and PBO trials at EE. BASE, baseline; R1, after 60 min of recovery; R2, after 120 min of recovery.

There were no significant differences between PBO and CHO for RPE (*P* = 0.473) or HR (*P* = 0.869), but, as expected, both increased over the duration of the exercise bout (*P* < 0.001). There was also no interaction between condition and time for either RPE (*P* = 0.847) or HR (*P* = 0.170). Blood glucose concentrations (Fig. 1) remained relatively unchanged during PBO feeding (BASE: 4.7 ± 0.3 mmol/l, EE: 4.8 ± 0.8 mmol/l, R1: 4.4 ± 0.4 mmol/l, R2: 4.3 ± 0.3 mmol/l) but were significantly elevated with CHO feeding (*P* = 0.002) at EE (6.3 ± 0.6 mmol/l) compared with BASE (4.9 ± 0.2 mmol/l). Blood glucose concentrations were significantly (*P* < 0.001) higher in the CHO trial than in the PBO trial at EE. Blood lactate concentrations (Fig. 1) increased significantly from BASE to EE (*P* = 0.002) before returning back toward BASE during recovery.

### Markers of Bone Metabolism

**Fig. 2.**
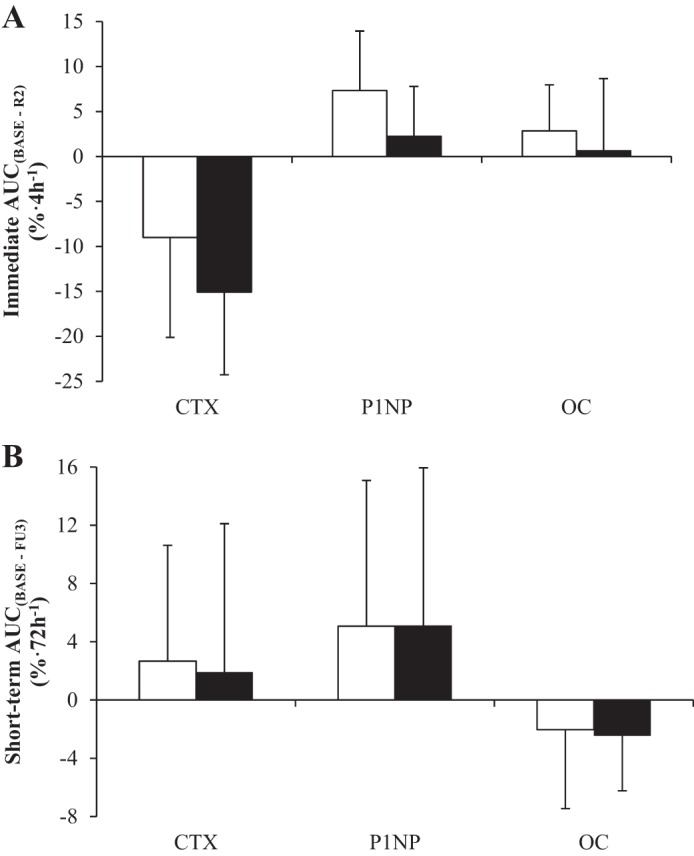
Immediate (*A*) and short-term (*B*) recovery areas under the curve (AUCs) for markers of bone resorption and formation on the CHO (■) and PBO (□) trials. CTX, COOH-terminal telopeptide region of collagen type 1; P1NP, NH_2_-terminal propeptides of procollagen type 1; OC, osteocalcin; FU3, 3rd follow-up day.

β-CTX increased by between 6% and 14% from BASE to the follow-up days in the PBO trial (BASE: 0.54 ± 0.14 ng/ml, FU1: 0.56 ± 0.14 ng/ml, FU2: 0.58 ± 0.19 ng/ml, FU3: 0.61 ± 0.16 ng/ml; Table 1). AUC analysis showed that the β-CTX response to exercise was significantly lower in the immediate postexercise period with CHO than with PBO (*P* = 0.028; Fig. 2*A*), although there was no difference in the short-term response over the follow-up days (*P* = 0.856; Fig. 2*B*).

On the follow-up days, P1NP was 5–14% (FU1: 67.9 ± 33.2 ng/ml, FU2: 66.2 ± 31.7 ng/ml, FU3: 68.4 ± 28.0 ng/ml) higher than at BASE (63.1 ± 30.5 ng/ml) in the PBO trial (Table 1). AUC analysis showed significantly lower P1NP concentrations in the CHO trial compared with the PBO trial in the hours following exercise (*P* = 0.021; Fig. 2*A*), but there were no differences between trials over the follow-up days (*P* = 0.721; Fig. 2*B*).

OC concentrations were not affected by exercise in the PBO trial (Table 1) or by CHO ingestion during exercise either in the immediate (*P* = 0.343; Fig. 2*A*) or short-term (*P* = 0.786; Fig. 2*B*) recovery periods.

### Modulators of Calcium Metabolism

PTH increased (*P* < 0.001) by 87% at EE (6.6 ± 2.0 pmol/l) compared with BASE (3.7 ± 1.2 pmol/l) in the PBO trial. Thereafter, concentrations returned toward BASE levels and were slightly lower than BASE at R1 (3.6 ± 1.2 pmol/l) and R2 (3.3 ± 1.3 pmol/l). PO_4_ was increased by 21% at EE (1.4 ± 0.1 mmol/l) because of the exercise bout (*P* < 0.001) and then returned to BASE values (1.1 ± 0.1 mmol/l) by R1 (1.1 ± 0.2 mmol/l). No exercise effect was shown for albumin-adjusted calcium (ACa) in the PBO trial (*P* = 0.871). There were no immediate or short-term effects of CHO feeding on calcium metabolism markers (Table 2).

**Table 2.** Immediate and short-term data for changes in modulators of bone and calcium metabolism during exercise

		Immediate Responses, %/4 h			Short-Term Responses, %/72 h		
	*n*	PBO	CHO	*P* value	PBO	CHO	*P* value
*Markers of calcium metabolism*							
PTH, pmol/l	10	9 ± 10	8 ± 13	0.801	1 ± 16	3 ± 19	0.812
PO_4_, mmol/l	8	1 ± 5	−2 ± 6	0.246	−6 ± 5	2 ± 9	0.040
ACa, mmol/l	8	0 ± 3	1 ± 3	0.624	0 ± 2	−2 ± 4	0.239
*Modulators of bone metabolism*							
Insulin, pmol/l	8	−15 ± 17	1 ± 26	0.112	22 ± 59	−3 ± 23	0.208
Cortisol, nmol/l	8	3 ± 19	0 ± 25	0.779	−2 ± 17	−5 ± 13	0.595
IL-6, pg/ml	8	625 ± 337	398 ± 384	0.036	−70 ± 230	−58 ± 143	0.327
Leptin, ng/ml	7	−14 ± 6	−12 ± 5	0.201	0 ± 1	2 ± 11	0.622
OPG, pmol/l	8	9 ± 26	25 ± 35	0.109	34 ± 80	26 ± 66	0.674
GLP-2, ng/ml	10	2 ± 10	6 ± 9	0.422	−2 ± 17	4 ± 21	0.508

Data are mean ± SD changes in concentration of modulators of bone and calcium metabolism with or without CHO supplementation during exercise.

### Other Modulators of Bone Metabolism

Significant effects of exercise (PBO trial) were shown for insulin (*P* < 0.001), IL-6 (*P* < 0.001), and leptin (*P* < 0.01), but there were no significant effects of exercise on cortisol, OPG, or GLP-2. CHO feeding significantly attenuated the elevation in IL-6 concentrations (223%) seen immediately after exercise in the PBO trial (Table 2; *P* = 0.036), although these differences did not persist over the follow-up days (*P* = 0.327). There were no other immediate or short-term effects of CHO feeding on any of the remaining modulators of bone metabolism measured (Table 2).

## DISCUSSION

Our main findings were that *1*) CHO feeding during exercise attenuated the β-CTX and P1NP responses in the hours but not days following exercise and *2*) IL-6 responded in a similar manner to bone turnover after CHO feeding during exercise.

The reduction in bone resorption with CHO feeding during strenuous exercise suggests a potential strategy for athletes and those performing arduous occupational training (e.g., the military recruit) to minimize increased bone resorption resulting from such exercise (23). From the results of the present investigation, it should be noted, however, that the magnitude of the effect of CHO feeding on β-CTX concentrations was relatively small, which in itself might not be that clinically significant. If these effects were repeatable over subsequent strenuous exercise bouts (as would be the case during athletic or military training programs), however, then there could be a physiological or clinical benefit. Future studies should determine the effect of repeated feeding during exercise across a training program on bone turnover. It should also be noted that CHO feeding attenuated the exercise-induced increase in P1NP, indicating that the dynamic balance between bone resorption and formation was maintained. The bone marker responses to CHO ingestion during exercise in the present study are similar to the responses observed by others at rest (3), where a reduction in β-CTX (18%) and P1NP (4%) in response to breakfast feeding, compared with fasting, was shown. While this is consistent with the present findings incorporating an exercise intervention, it does not concur with our previous study (22), which showed that resting concentrations of β-CTX, but not P1NP or OC, were reduced after a preexercise mixed meal compared with fasting.

One possible explanation for the immediate effects of CHO feeding on the bone resorption response to exercise is the concomitant reduction in the IL-6 response, with a strong correlation (*r* = 0.74, *P* < 0.05) existing between the immediate responses of IL-6 and β-CTX in the CHO trial. The ingestion of CHO before and during endurance exercise attenuates the rise in circulating IL-6 associated with exercise (15). Starkie et al. (24) showed that a total ingestion of 64 ± 3 g of CHO before and during 60 min of running and cycling attenuated the rise in plasma IL-6 associated with both modes of exercise. Increases in circulating cytokines (16, 17) have been shown in response to heavy or unaccustomed exercise and might have a regulatory function in bone metabolism (13), potentially providing a mechanism that underpins the observed short-term effect of CHO ingestion during exercise on bone metabolism. Evidence from in vitro and animal models suggests that IL-6 is an activator of osteoclastogenesis and bone resorption (12). It can stimulate osteoclast differentiation but can also, in the presence of soluble IL-6 receptors (sIL-6Rs), stimulate osteoclast activity (12). Palmqvist et al. (18) have shown that IL-6, when combined with its soluble receptor, stimulates bone resorption, as well as mRNA and protein expression of receptor activator of nuclear factor κB ligand (RANKL) and OPG in murine calvarial bone. sIL-6R can bind its ligand and induce cellular responses through association with the glycoprotein 130 receptor subunit (gp130), thus acting as an IL-6 agonist. IL-6 stimulates gp130 on stromal or osteoblastic cells, subsequently resulting in downstream signal transducer and activator transcription 3-mediated expression of RANKL and stimulation of osteoclast formation. In addition, others (20) have suggested the presence of a T-cell cytokine that can stimulate IL-6 in human osteoblastic cells. While this indicates a potential mechanism for the present findings, it should be noted that there was no effect of CHO feeding on circulating OPG concentrations and we cannot confirm the circulating sIL-6R or RANKL concentrations in the present study. Previous studies have suggested an effect of exercise on sIL-6R concentrations (for review see Ref. 19), but there is no study, to our knowledge, that has reported the effects of CHO feeding. The measurement of circulating RANKL concentrations was not possible in the present study because of the lack of a suitable assay. In addition, when measuring the circulating concentrations of molecules it is not possible to be certain of the biological actions occurring in particular tissues.

There was a significantly attenuated P1NP response in the hours after exercise in the CHO trial compared with the PBO trial, suggesting that CHO feeding during prolonged exercise affects bone formation in addition to bone resorption. There was no concomitant effect on circulating OC concentrations, which is inconsistent with the P1NP and IL-6 responses to CHO ingestion during exercise in the present study, since some have suggested that it is a marker of coupled bone turnover (9), is capable of suppressing IL-6 release (8), and has importance in the relationship between bone remodeling and energy metabolism (6).

The response of P1NP in concert with the effect shown on β-CTX would suggest that CHO feeding during exercise reduced overall bone turnover in the hours following exercise but the balance between resorption and formation was maintained. While a reduction in IL-6 release during exercise with CHO feeding is a plausible mechanism to explain the observed reduction in β-CTX concentrations, the reason for the attenuated postexercise P1NP concentrations with CHO feeding during exercise is currently unknown but is likely to involve cellular cross talk between the osteoclasts and osteoblasts, given their very close association.

Potential mechanisms for the suppression of bone turnover by CHO feeding during exercise relate to effects on PTH or on the incretin and enteric hormones. Bone turnover was decreased along with PTH in a previous study employing the hypoglycemic clamping technique (4), suggesting that short-term alterations to bone turnover are due to direct effects of hypoglycemia (glucose concentrations were clamped at 2.5 mmol/l for 105 min) on bone cells or are mediated by changes in regulatory hormone concentrations triggered by hypoglycemia (4). The present study did not show any immediate effect of CHO provision during exercise on the circulating concentrations of PTH, ACa, or PO_4_, making it unlikely that the effects of CHO supplementation during exercise on bone turnover were mediated by alterations in calcium metabolism. CHO did not significantly alter the immediate or short-term responses of leptin, insulin, cortisol, or GLP-2 to exercise, indicating that alterations in these hormones were also unlikely to be responsible for the effect of CHO supplementation on bone turnover. Despite the fact that the present study involved 120 min of running at 70% V̇o_2 max_, blood glucose concentrations remained relatively stable, even when participants were not fed CHO in the PBO trial, with blood glucose concentrations not reaching hypoglycemic levels.

Given that the data from the PBO trial show a 5–15% increase in β-CTX during the follow-up days compared with BASE, and others (23) also report greater and more prolonged increases in β-CTX following exhaustive exercise, it is clear that CHO feeding during exercise alone might not be sufficient to result in a more prolonged or greater suppression of the increase in bone resorption. This might be because the amount of CHO provided (102.1 ± 10.6 g) during exercise was not sufficient to prolong the effect observed during the immediate recovery period, possibly because the CHO was used by the muscle to support glycogen resynthesis. Another possibility is that participants resumed their habitual diet once leaving the laboratory on *day 4*, meaning that any extra CHO provided during exercise in the CHO trial would become less meaningful as a proportion of the total daily energy intake. As such, the clinical implications of the present study findings remain unclear; future work could explore higher CHO intakes during exercise or the maintenance of high CHO intakes in the days following exercise for skeletal benefits. The amount of CHO provided during exercise in the present study (∼50 g/h) is consistent with the amount and rate typically recommended for endurance exercise benefits (10), although in current practice athletes typically consume 60–70 g/h (11) and athletes performing prolonged exercise (e.g., triathletes) are advised to increase their CHO intake (from multiple CHO sources) to 80–90 g/h (11).

In conclusion, CHO supplementation during prolonged running reduced bone turnover in the hours following exercise. A possible mediator of the immediate bone resorption response to exercise when CHO was fed during exercise was IL-6. The mechanism underlying the reduced P1NP response remains unknown. No changes in markers of calcium metabolism or incretin and enteric hormone concentrations were observed with CHO feeding, suggesting that they are unlikely mediators of the effect of CHO on bone turnover.

## GRANTS

This work was funded by the UK Ministry of Defence (Army).

## DISCLOSURES

No conflicts of interest, financial or otherwise, are declared by the author(s).

## AUTHOR CONTRIBUTIONS

Author contributions: C.S., W.D.F., and J.P.G. conception and design of research; C.S., I.V., and T.W.J. performed experiments; C.S., I.V., R.M.J., and J.C.T. analyzed data; C.S., I.V., T.W.J., and R.M.J. interpreted results of experiments; C.S. drafted manuscript; C.S., I.V., T.W.J., R.M.J., W.D.F., and J.P.G. edited and revised manuscript; C.S., I.V., T.W.J., R.M.J., J.C.T., W.D.F., and J.P.G. approved final version of manuscript; R.M.J. prepared figures.
